# Supplementary material for: Risk prediction of all-cause mortality in hospitalized patients with severe acute pancreatitis by serum urea nitrogen/albumin ratio
Source: PLoS One. 2025 Sep 19;20(9):e0326543. doi: 10.1371/journal.pone.0326543 (PMC12448361; doi:10.1371/journal.pone.0326543)
Supplement: Attachment — Submitted filename: Response to reviewer.docx [file pone.0326543.s002.docx]

Dear Editor and Reviewer:

On behalf of all the contributing authors, I would like to express our sincere appreciations of your letter and reviewers’ constructive comments concerning our article entitled “Risk prediction of all-cause mortality in hospitalized patients with severe acute pancreatitis by serum urea nitrogen/albumin ratio” (Manuscript No: PONE-D-24-51800). These comments are all valuable and helpful for improving our article. According to the Editor and reviewers’ comments, we have made extensive modifications to our manuscript to make our results convincing. In this revised version, changes to our manuscript were all highlighted within the document by using red-colored text. Point-by-point responses to the nice Editor and reviewer are listed below this letter.

Editor

Response

Thank you very much for the suggestion, we think it's a very good one. We carefully reviewed the literature and made changes to the discussion and formatting sections of the revised manuscript. Hope to get your approval!

Reviewer 2

Comment 1：

Results can include numerical values, percentages, and other interesting statistical baseline characteristics for the study group, mean age of the study group, gender distribution, significant comorbidities, drug use, etiology, outcomes, complications of pancreatitis, and other values of interest in acute pancreatitis, such as blood glucose levels, calcium levels, SOFA scores, if included in the manuscript, It will be easier for the reader to read and absorb than to refer to the table at every point.

In the results, the authors can discuss which factors differ significantly and provide numerical values so that the reader can see them.

Response

Thank you very much for your suggestion,We think this is an excellent suggestion. We carefully reviewed the literature and made changes to the discussion section of the revised manuscript. Details have been added in P12-13, Line 239~264.

Comment 2：

Authors can also discuss in the results whether they found other interesting factors in the study that might be interesting to the reader and lead to future research.

Response

Thank you very much for your suggestion,We think this is an excellent suggestion. We carefully reviewed the literature and made changes to the discussion section of the revised manuscript. Details have been added in P16, Line 325~330.

Comment 3：

The authors could discuss the results under Models 2 and 3, specifically Model 3, by mentioning clinical expertise to clarify what they indicate and how this knowledge was derived from scientific interest.

The authors can mention and explain in the results the factors selected by the Baruta and SHAP algorithms.

Interested authors will explain the importance of the Z-score, SHP and BAR in the BAR plot, there are two groups of icons, 0, 1 but it is not clear what is 0 and what is 1, the author can annotate the plot to be clearer.

The plots of HR are represented as forest plots on the chart, but usually forest plots are usually represented by 0 and values on both sides, for the sake of meaning, the plots here are represented by 1, so the reader may feel that these values are not important, but in fact they are not important, can the author look into this issue? If they don't want to change the current presentation, they can explain the same thing in the results so that the reader will be clearer.

Response

Thank you very much for your suggestion,We think this is an excellent suggestion. We carefully reviewed the literature and made changes to the discussion section of the revised manuscript. The selection of covariates for adjustment in our model was informed by a systematic review of existing literature on mortality risk factors in acute pancreatitis, supplemented by feature importance analyses using SHAP (SHapley Additive exPlanations) and Boruta machine learning algorithms. This dual approach ensured that variables were prioritized based on both evidence from prior studies and data-driven predictive relevance.and we use R to calculate, and the HR we calculate is greater than 1, so we use starting from 1 in the forest graph.and In the early stage, we read a large number of relevant literature and summarized the relevant important variables.Following the determination of the relevance of the study variables, feature selection was a crucial step in reducing the number of features in the large dataset. A crucial method of feature selection was the Boruta algorithm, which was based on the random forest classifier method.Details have been added in P27 Line 564-566 and P11, Line 213~226.

Comment 4：

It would be helpful if the authors could mention the analysis subgroup they chose and the rationale behind their methodological choice of the analysis subgroup. The authors can mention the values of unimportant subgroups in the results because only the important factors are mentioned in the results.

Response

Thank you very much for your suggestion,We think this is an excellent suggestion. We carefully reviewed the literature and made changes to the discussion section of the revised manuscript. Details have been added in P15-16, Line 306~317 and P10-11, Line 200~211.

Comment 5：

The authors excluded patients with HIV, kidney disease, and malignancies, which I think may be due to the fact that the patients are more severely ill and have lower albumin. Could the author please clarify this in the manuscript as it is valuable to the reader.

Response

Thank you very much for your suggestion,We think this is an excellent suggestion. We carefully reviewed the literature and made changes to the discussion section of the revised manuscript. Details have been added in P5, Line 86~94.

Comment 6：

During the discussion, authors may discuss the results of their study, as well as the similarity of the baseline characteristics of their study arm to other pancreatitis studies, so that the reader can identify the applicability of the study in clinical practice.

The authors could discuss the different values of BAR in various diseases, rather than discussing its importance in other diseases in general, and limit it to pancreatitis-like diseases, such as those with multiple organ dysfunction, such as sepsis.

The authors can discuss the BAR and cut-offs used in pancreatitis, as well as the reasons behind the cut-offs, so that it is clear to the reader.

It is expected that it will be resubmitted once the recommendations have been implemented.

Response

Thank you very much for your suggestion,We think this is an excellent suggestion. We carefully reviewed the literature and made changes to the discussion section of the revised manuscript. Details have been added in P13-14, Line 269~283 and P7, Line 134~139.

Reference：Since the 40th reference in the original manuscript was required for the revision of the reviewer's comments, this reference has been placed in the 28th paragraph of the revised manuscript.

We tried our best to improve the manuscript and made some changes We appreciate for Editor and Reviewers’ warm work earnestly, and hope the correction will meet with approval. Once again, thank you very much for your comments and suggestions.

Kind regards,

Ziwen Lv
